# Modulation of transcriptional activity in brain lower grade glioma by alternative splicing

**DOI:** 10.7717/peerj.4686

**Published:** 2018-05-14

**Authors:** Jin Li, Yang Wang, Xianglian Meng, Hong Liang

**Affiliations:** College of Automation, Harbin Engineering University, Harbin, Heilongjiang, China

**Keywords:** Alternative splicing, Amyloid precursor protein, EST domain-containing proteinElk-1, Serine/threonine kinase 16, Modulator, Lower grade glioma

## Abstract

Proteins that modify the activity of transcription factors (TFs) are often called modulators and play a vital role in gene transcriptional regulation. Alternative splicing is a critical step of gene processing, and differentially spliced isoforms may have different functions. Alternative splicing can modulate gene function by adding or removing certain protein domains and thereby influence the activity of a protein. The objective of this study is to investigate the role of alternative splicing in modulating the transcriptional regulation in brain lower grade glioma (LGG), especially transcription factor ELK1, which is closely related to various disorders, including Alzheimer’s disease and Down syndrome. The results showed that changes in the exon inclusion ratio of proteins APP and STK16 are associated with changes in the expression correlation between ELK1 and its targets. In addition, the structural features of the two modulators are strongly associated with the pathological impact of exon inclusion. The results of our analysis suggest that alternatively spliced proteins have different functions in modifying transcription factors and can thereby induce the dysregulation of multiple genes.

## Introduction

Alternative splicing (AS) is a key regulator of gene expression as it generates numerous transcripts from a single protein-coding gene. In humans, more than 95% of multi-exonic protein-coding genes undergo AS (Wang et al., 2008), and AS plays an important role in cellular differentiation and organism development (Castle et al., 2008; Wang et al., 2008). As AS affects numerous genes and is highly important for regulating the normal expression and tissue specificity of a given gene, it is not surprising that changes in AS are frequently associated with human disease, such as cancers (Kozlovski et al., 2017) and neurodegenerative diseases (Scotti & Swanson, 2016). Recent genome-wide analyses of cancer transcriptomes have demonstrated that splicing changes are often global rather than gene specific (Jung et al., 2015). Undoubtedly, widespread splicing changes, such as altered cassette exon inclusion ratios of proteins, influence the expression of numerous genes and consequently cause aberrant gene regulation.

Lower grade glioma (LGG) is a type of cancer that develops in the glial cells of the brain. Tumors are classified into grades I, II, III or IV based on standards set by the World Health Organization (Ostrom et al., 2013). Regardless of tumor grade, tumors compress normal brain tissue as they grow, frequently causing disabling or fatal effects. The Cancer Genome Atlas (TCGA) consortium has produced a comprehensive somatic landscape of glioblastoma by combining molecular and clinical data that have become a valuable resource for studying gene deregulation in LGG.

Modulators are proteins that modify the activity of transcription factors (TFs) and influence the expression of their target genes. Our current knowledge of TF modulation mainly comes from experimental studies that measure the expression levels of a few target genes (Lachmann et al., 2010). The objective of this study is to explore the role of AS in modulating the transcriptional activities of TFs in LGG. The modulated relationships among TF-modulator-targets are inferred using a known probabilistic model named GEM (Babur et al., 2010). EST domain-containing protein Elk-1 (ELK1) is one TF whose regulation activity is most influenced by 162 splicing events corresponding to 123 AS modulator proteins. Finally, amyloid precursor protein (APP) and serine/threonine kinase 16 (STK16), modulators whose exon inclusion ratios are associated with the activity of ELK1, are analyzed in detail.

## Materials and Methods

### Construction of triplets

We implemented the GEM algorithm (Babur et al., 2010) to predict (splicing modulator-TF-target) triplets. There are four input types: gene expression profiles, gene splicing profiles, modulator list and TF-target relations. The modulator hypothesis predicts that the correlation between the expression levels of the TF and the target must change as the splicing level of the modulator changes. The percentage of exon inclusion ratio (PSI) is used to estimate the splicing level of a candidate modulator in LGG. We established a 5% false discovery rate as the threshold to call the triplets.

### Data processing and selection

**Figure 1:**
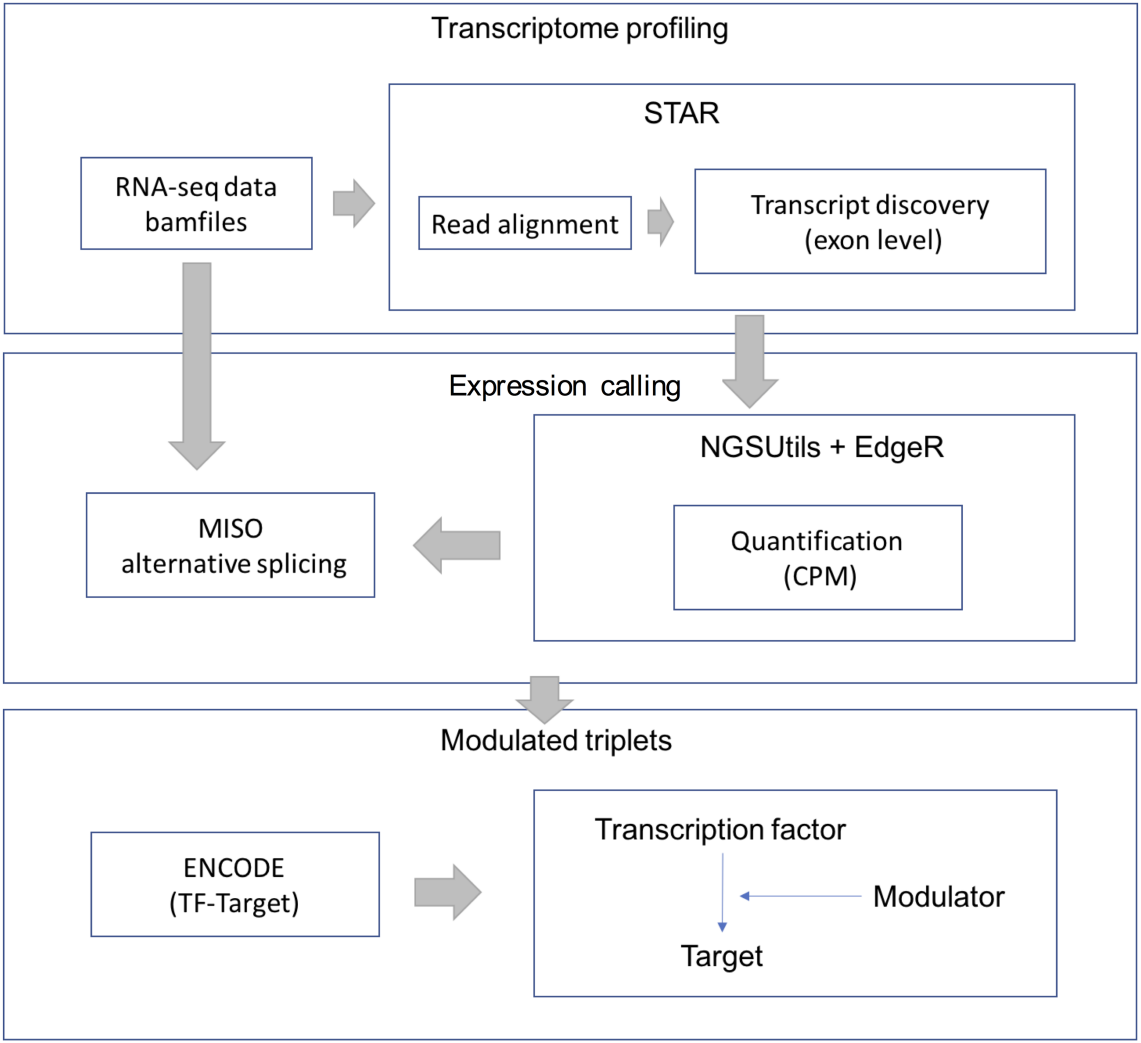
Workflow for data processing and selection. The whole workflow including three parts: obtain the transcriptional profile, expression and splicing calling and construct the modulated triplets.

RNA-Seq data were downloaded from the TCGA-LGG data portal as bam files. STAR aligner (version 2.3.0) was used to align each file uniquely to the hg19 human genome. We retained uniquely aligned reads with a minimum splice junction overhang of five nucleotides using default parameters. The gene expression level was estimated using the NGSUtils tool (version 0.5.9) (Breese & Liu, 2013) with default parameters for calling gene expression. The splicing level (PSI) was estimated using a probabilistic model called Mixture of Isoforms (MISO) (Katz et al., 2010). The TF-target relations were derived from the ENCODE (The Encyclopedia of DNA Elements) project. The workflow of data processing and selection is described in Fig. 1.

For the candidate modulators, we keep the splicing events where over 95% samples have confidence interval (CI) less than 0.25 and only analyze predicted cassette exons that have at least 10 reads supporting exon inclusion or exclusion in at least one sample. We fill the missing PSI value of a sample with the median PSI value of that splicing event. Finally, AS events were selected based on candidate modulators whose PSI IQR (interquartile range) were larger than 0.1 As the input data require sufficient variability, we filtered out genes whose gene expression coefficient variation (CV) was less than 50% and kept genes in which over 95% of samples had expression values.

### Database and related software

The implementation of GEM is available through SourceForge (https://sourceforge.net/projects/modulators). Statistical analysis and data processing were performed using R version 3.0.1 (http://www.r-project.org). DAVID (Dennis Jr et al., 2003) and IPA (Ingenuity Pathway Analysis) were used to perform gene function and pathway analysis. Protein-protein interactions were predicted by the STRING database (http://string-db.org).

## Results

### Global inferring modulators of all TFs

**Figure 2:**
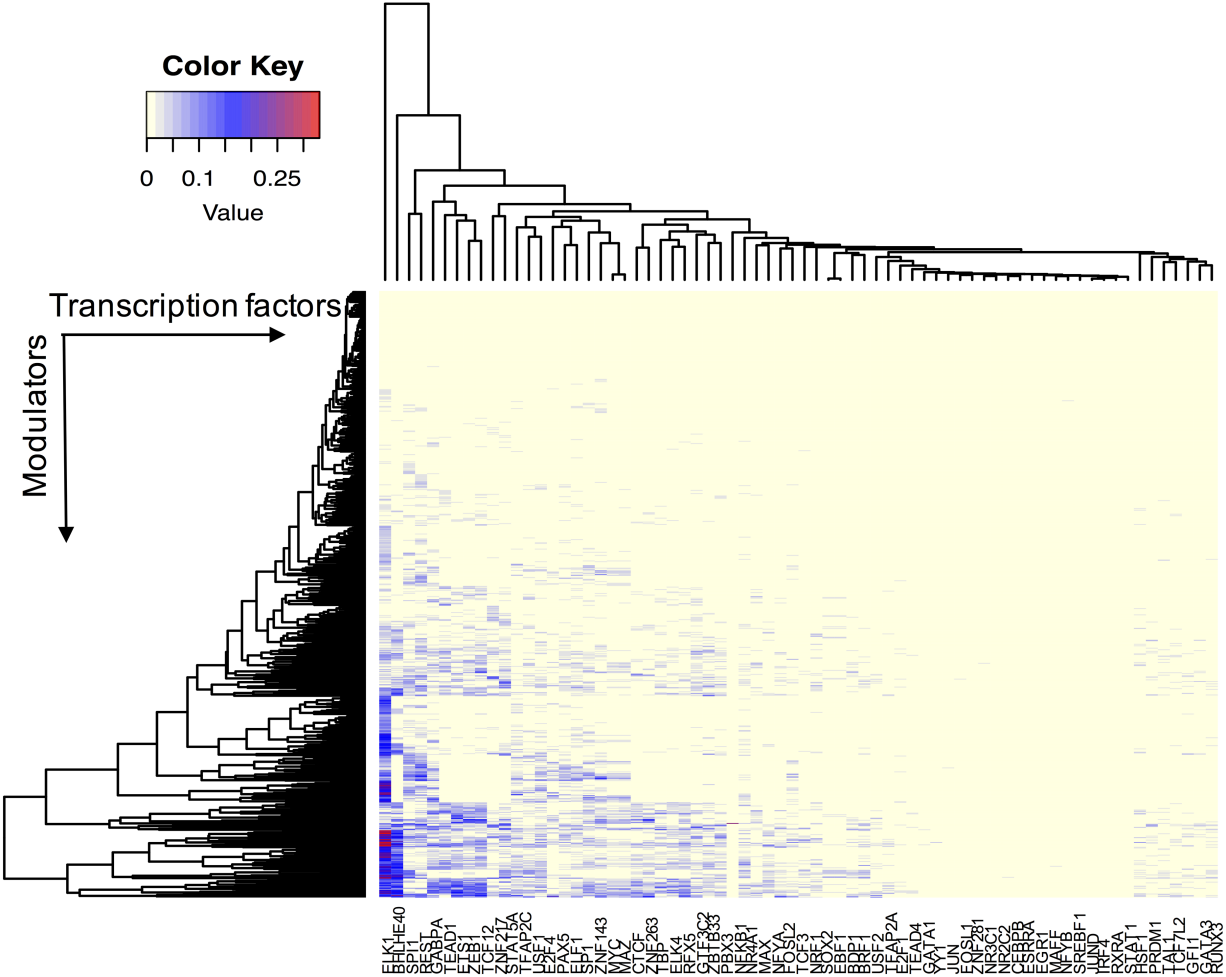
The effect of transcription factor activity regulated by splice modulator proteins. Each row represents a candidate modulator and each column indicates a transcription factor. The much darker color means a much higher percent target of TF is influenced.

We assume that all TFs have the potential ability to interact with their modulator candidates. Seven hundred and sixty-five AS events were considered putative modulators, and 173,598 TF-target pairs composed of 74 TFs and 17,425 targets were used to infer modulated triplets. The number of inferred splicing modulators varied across all TFs, and the percent of influenced targets ranged from 0 to 33.5% for each TF (Fig. 2).

**Figure 3:**
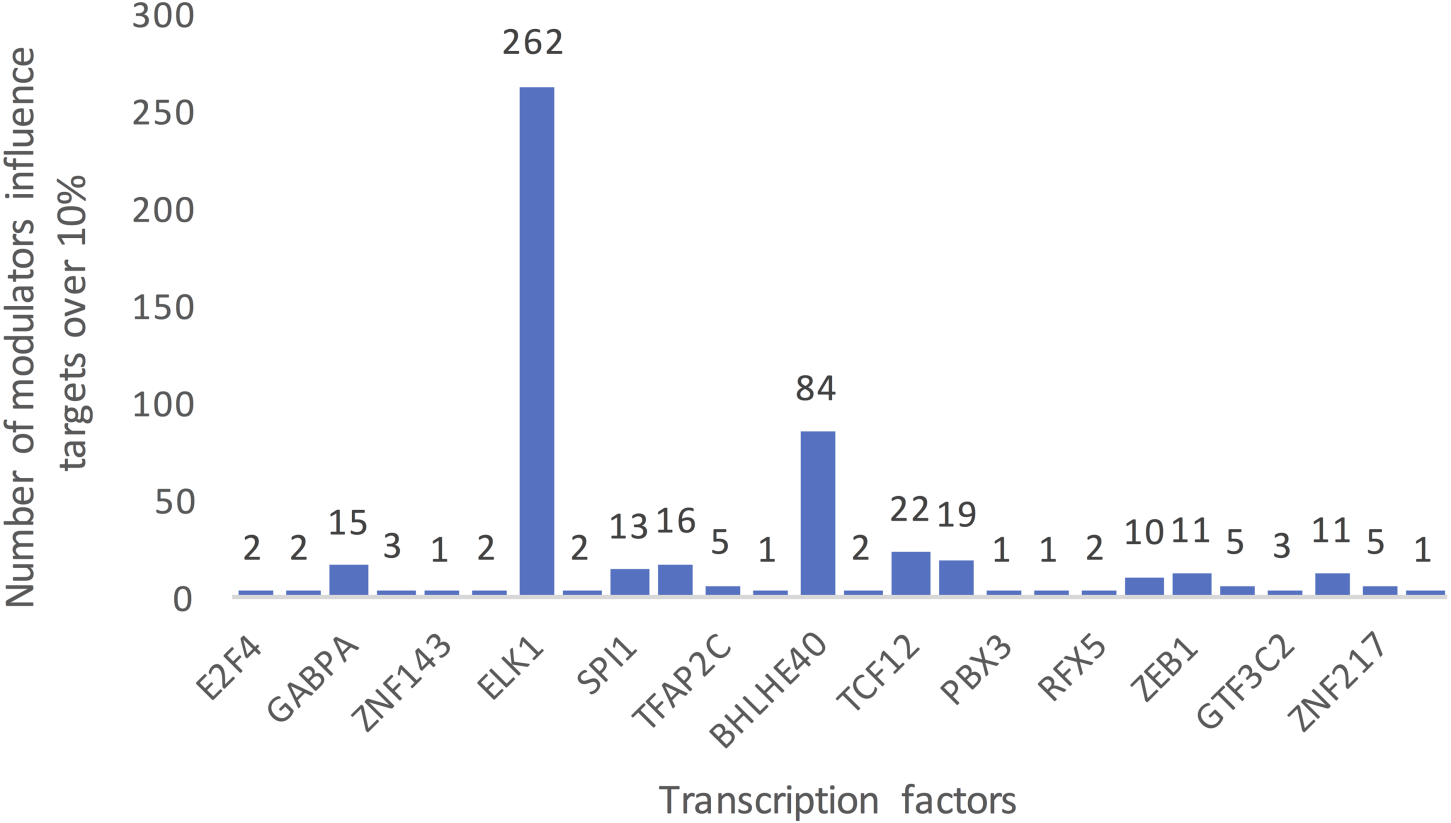
Summarized counts of inferred modulators of TFs. The *c*-axis represents the transcription factor list, and the *y*-axis represents the counts of inferred modulators. The number on each TF indicates the number of modulators of each TF that influence more than 10% of its targets.

Figure 3 summarizes the number of modulators of 26 TFs whose influence targets over 10%. The number of inferred modulators ranges from 1 to 262. EST domain-containing protein Elk-1 (ELK1) was one of the 26 TFs that had the greatest number of predicted modulators. A total of 262 splicing events corresponding to 187 proteins were identified as ELK1 modulators because their splicing outcomes highly correlated with changes in the transcriptional activity of ELK1.

### Gene function analysis of ELK1 modulators in LGG

ELK1 is a member of the ETS TF family, which is closely related to various disorders, including Alzheimer’s disease, Down syndrome and breast cancer, in a dose-dependent manner (Peng et al., 2017). ELK1 is a member of the Ets (T twenty-six) oncogene family of TFs, which includes nuclear phosphoproteins involved in many biological processes, such as cell growth, survival hematopoiesis, wound healing, cancer and inflammation (Sharrocks, Yang & Galanis, 2000; Besnard et al., 2011). In addition, ELK1 can significantly regulate the expression of c-Fos, which is a key gene for cell proliferation and differentiation (Chambard et al., 2007). In this study, we inferred 540 splicing events as ELK1 modulators.

**Figure 4:**
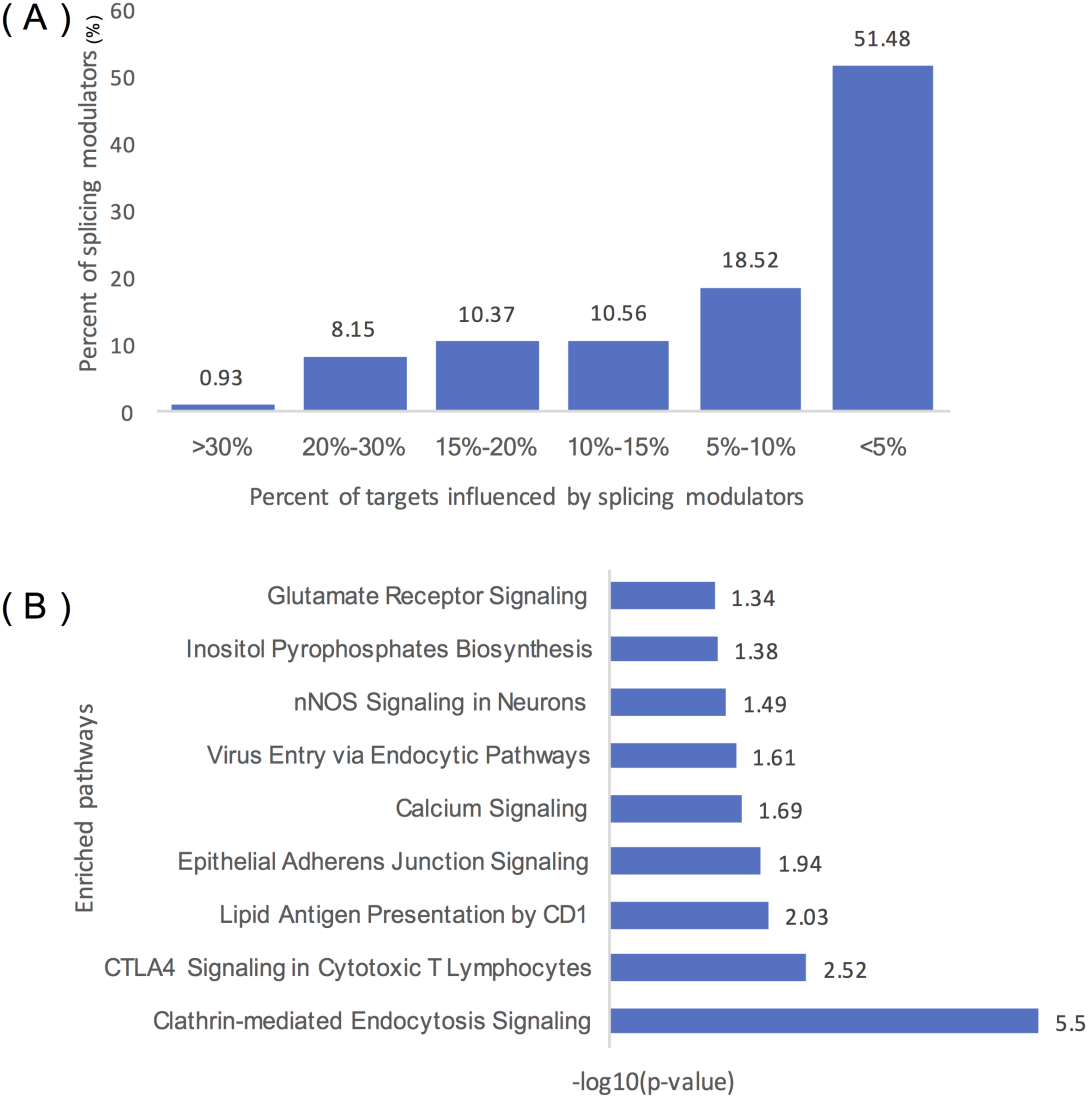
Statistical analysis of the modulators of ELK1. (A) Distribution of the number of ELK1 modulators. The *x*-axis represents the percentage of targets influenced by modulator proteins. The *y*-axis indicates the percent of modulators of ELK1. The number noted on each column indicates the percent of modulators in each classification. (B) IPA of ELK1 modulators that influenced over 10% of its targets. The *x*-axis is the −log10 transformed *p* of each enriched pathway (*y*-axis).

Figure 4A summarizes the distribution of inferred modulators of ELK1. Two hundred and sixty-two modulators influence over 10% of ELK1’s targets, 49 modulators influence at least 20% of its targets, and five modulators influence more than 30% of its targets, including ‘chr2:39931221:39931334: +@chr2:39934189:39934326: +@chr2:39944150:39945104: +’ (TMEM178A, Transmembrane protein 178A precursor); ‘chr2:74685527:74685798: +@chr2:74686565:74686689: +@chr2:74686770:74686872: +’ (WBP1, WW domain binding protein 1); ‘chr2:36805740:36806008: -@chr2:36787928:36788008: -@chr2:36785581: 36785656: -‘ (FEZ2, Fasciculation and elongation protein zeta-2); and ‘chr5:175788605: 175788809: -@chr5:175786484:175786570: -@chr5:175782574:175782752:’ (KIAA1191, Putative monooxygenase p33MONOX), ‘chr2:74685527:74685798: +@chr2:74686565: 74686679: +@chr2:74686770:74686872: +’ (WBP1).

As many inferred modulators may have similar or related protein functions, we performed pathway and function enrichment analysis to explore the functions of these modulated genes. We filtered modulators that influenced less than 10% of the targets, and 262 splicing events as modulators corresponding to 129 proteins remained as ELK1 final modulators. After removing duplicated gene symbols and unannotated genes, 126 proteins were mapped to the Ingenuity Knowledge Base and subjected to core analysis.

The results showed that more than 80% of the splicing proteins related to cancer were enriched, and most of the enriched canonical pathways overlapped with certain genes. As summarized in Table 1, these modulators were enriched in three types of disease: neurological disease, organismal injury and abnormalities disease, and cancer. Molecular and cellular function enrichment analysis showed that more than 20% of the modulators were associated with cellular movement (28/123), cellular assembly and organization (32/123), and cellular function and maintenance (26/123); 11% and 8% of the modulators were highly enriched in cell morphology (14/123), and cell-to-cell signaling and interaction process (10/123), respectively. The top five modulator-enriched pathways (Fig. 4B) were highly (*p* < 0.05) associated with signaling processes, including clathrin-mediated endocytosis signaling, CTLA4 signaling in the cytotoxic T lymphocyte pathway, nNOS signaling in neurons, and calcium signaling pathways.

**Table 1:** ELK1 modulators protein function and disease enrichment (*p* < 0.001). Each number in the table indicates the account of ELK1 modulators enriched in specific function or disease. The statistical threshold is *p* < 0.001.

**Molecular and Cellular Functions**			
Cellular assembly and organization	32	Cellular movement	28
Cellular function and maintenance	26	Cell morphology	14
Cell-to-cell signaling and interaction	10		
**Diseases and Disorders**			
Neurological disease	37	Organismal injury and abnormalities disease	113
Cancer	110		

### APP modulates ELK1 transcriptional activity

Amyloid precursor protein (APP) is one of the modulators of interest, and its analysis is described in detail here. An interaction between APP and ELK1 is mentioned in the STRING database. Several AS isoforms of APP have been observed in humans. The isoforms range in length from 639 to 770 amino acids, and certain isoforms are preferentially expressed in neurons; changes in the neuronal ratio of these isoforms have been associated with Alzheimer’s disease (Matsui et al., 2007).

One splicing event of APP detected as a modulator was “chr21:27354657:27354790:- @chr21:27372330:27372497:-@chr21:27394156:27394358:-”. Different inclusion ratios of the alternatively spliced exon in APP protein influence 18.6% of the targets of ELK1, and the seventh exon, which contains a vital domain named BPT/Kunitz inhibitor (BPTI) (residues 291–341), is the alternatively spliced exon. The splice isoforms that contain the BPTI domain possess protease inhibitor activity.

According to the GEM algorithm, unmodulated ELK1 activity was classified into three categories according to the value of α_f_: activation if positive, inhibition if negative, and inactive if zero. Similarly, by comparing α and β coefficents, modulators were classified into three classes: enhancing, attenuating or inverting the activity of ELK1. Hence, there are six possible categories of action. The APP modulation categories and their interpretations are listed in Table S1.

As summarized in Table 2, without APP modulation of ELK1, unmodulated ELK1 inhibits 172 targets and activates 31 targets. However, when APP interacts with ELK1 as a modulator, the original transcriptional activity of ELK1 changes: APP attenuates the inhibitory role of ELK1 for 164 targets, inverts its inhibitory activity for eight targets, and enhances activation for 14 targets. APP also dysregulates ELK1 activity on 31 targets by inverting the activity for one target and attenuating the activity of 30 targets.

**Table 2:** Interpretation of the categories of APP modulation, and the inequality constraints that the category should satisfy. Each number in the table indicates the number of triplets in each classification.

Modulation classification	Explanation	#triplets	*γ*	*α*_*f*_	*β*_*f*_	*β*_*m*_	*α*_*f*_ + *β*_*m*_
Attenuates inhibition	F, alone, inhibits T-M attenuates F activity	164	+	−			
Enhances inhibition	Modulated F inhibits T	0	−		−	−	−
Inverts inhibition	F, alone, inhibits T-M inverts F activity	8	+	−	+	+	+
Inverts activation	F, alone, activates T-M inverts F activity	1	−	+	−	−	−
Enhances activation	Modulated F activates T	14	+		+	+	+
Attenuates activation	F, alone, activates T-M attenuates F	30	−	+			

**Notes.**

‘ +’ and ‘ −’ signs in the columns indicate significantly positive and negative values, respectively.

We randomly selected four targets (ANKRD34A, DDX27, DVL3 and HEATR1) of ELK1 to explore the different activities of ELK1 under the modulation of differential inclusion levels of APP protein. Ideally, the inclusion level of the splicing modulator and expression of ELK1 should have high variance and low correlation in the samples. We divided rank-ordered PSI values of APP splicing modulators, extracting ELK1 and its target samples that were consistent with APP splicing modulator samples in upper/lower tertile. We then estimated the differences in correlation between ELK1 and its target using Spearman’s correlation.

**Figure 5:**
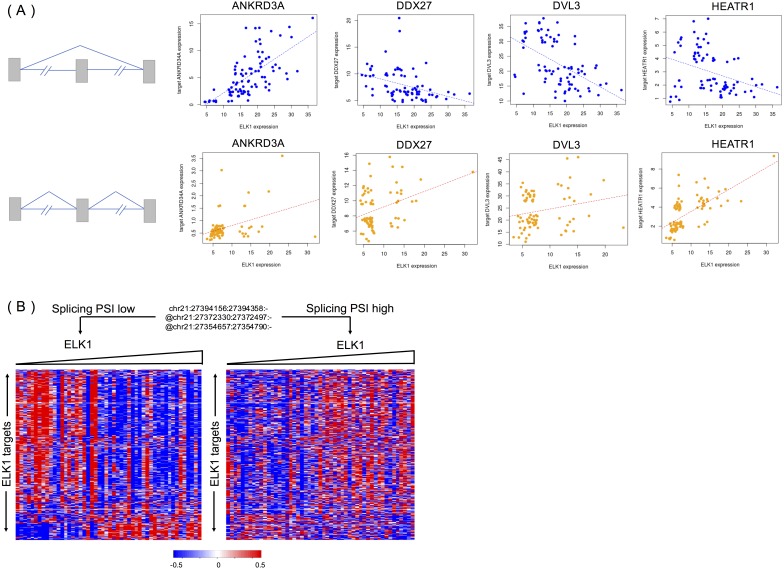
APP is a modulator that influences the activity of ELK1 in LGG. (A) Examples of different correlations between ELK1 and its targets under the modulation of APP with differential splicing levels. (B) Visualization of how APP regulates the stability of ELK1 protein. Gene expression profiles are displayed with genes in rows and samples in columns. Expression values of each gene are rank transformed, median centered and rescaled between (−0.5, 0.5). Samples were partitioned based on the alternatively spliced exon inclusion level of APP and sorted by the expression levels of ELK1 within each partition.

Figure 5A shows examples of APP-modulated ELK1 target genes and the corresponding action modes. As shown in Fig. 5A, when the exon inclusion level of APP was in the lower tertile, an increase in the gene expression level of ELK1 resulted in a significant increase in the gene expression of its target ANKRD34A. Spearman’s correlation of gene expression between ELK1 and ANKRD34A was 0.71 (*p* < 2.2*e* − 16), which means that in this condition, ELK1 plays an enhancement role on its target. However, when the PSI value of the APP modulator is in upper tertile, the correlation decreased into 0.30 (*p* = 0.0085). For the other two targets, DDX27 and DVL3, the correlations changed from −0.47 and −0.53, respectively, to non-significant (*p* > 0.1). For these three cases, the APP modulator attenuates the activation of ELK1. The opposite modulation occurs on target HEATR1. When the exon is spliced out of the protein, ELK1 negative regulates the expression of HEATR1 with a correlation as −0.38 (*p* = 0.0005); however, when the exon is excluded from the mature mRNA, the APP modulator inverts the activation of ELK1 on its target with a correlation of 0.70 (*p* < 2.2 − 16).

We evaluated the exon’s impact on APP protein using ExonImpact (Li et al., 2017b). The results showed that the alternatively spliced exons of APP protein that we detected have a high probability (0.57 and 0.48) of being associated with disease. This result indicates that changes in the inclusion or exclusion level of spliced exons can lead to significant changes with respect to APP protein function.

Figure 5B visualized the global effect of changing the inclusion ratios of alternately spliced exons in APP and influences on the relationship between ELK1 and its target. The two groups of samples are selected based on the seventh exon inclusion ratio of APP. The high and low inclusion groups contain samples with the top and bottom 30% of PSI values. The correlation patterns between ELK1 and its targets in the two groups are different, clearly showing that different splicing levels of APP can modulate the transcriptional activity of ELK1.

### STK16 modulates ELK1 transcriptional activity

The AS event “chr2:220111379:220111598:+@chr2:220111835:220111968:+@chr2: 220112137:220112257:+” for protein serine/threonine kinase 16 (STK16) is another interesting modulator that we identified. The inferred STK16-modulated triplets and their modulation categories are listed in Table S2 . STK16 is a membrane-associated protein kinase that phosphorylates on serine and threonine residues. An interaction between STK16 and ELK1 is inferred from the biochemical effect of one protein on another in the BioGrid database. The alternatively spliced exon that acts as a modulator of SKT16 is the 4th exon and is located in a region that encodes a kinase domain named Pkinase that is associated with the protein’s proton acceptor.

**Figure 6:**
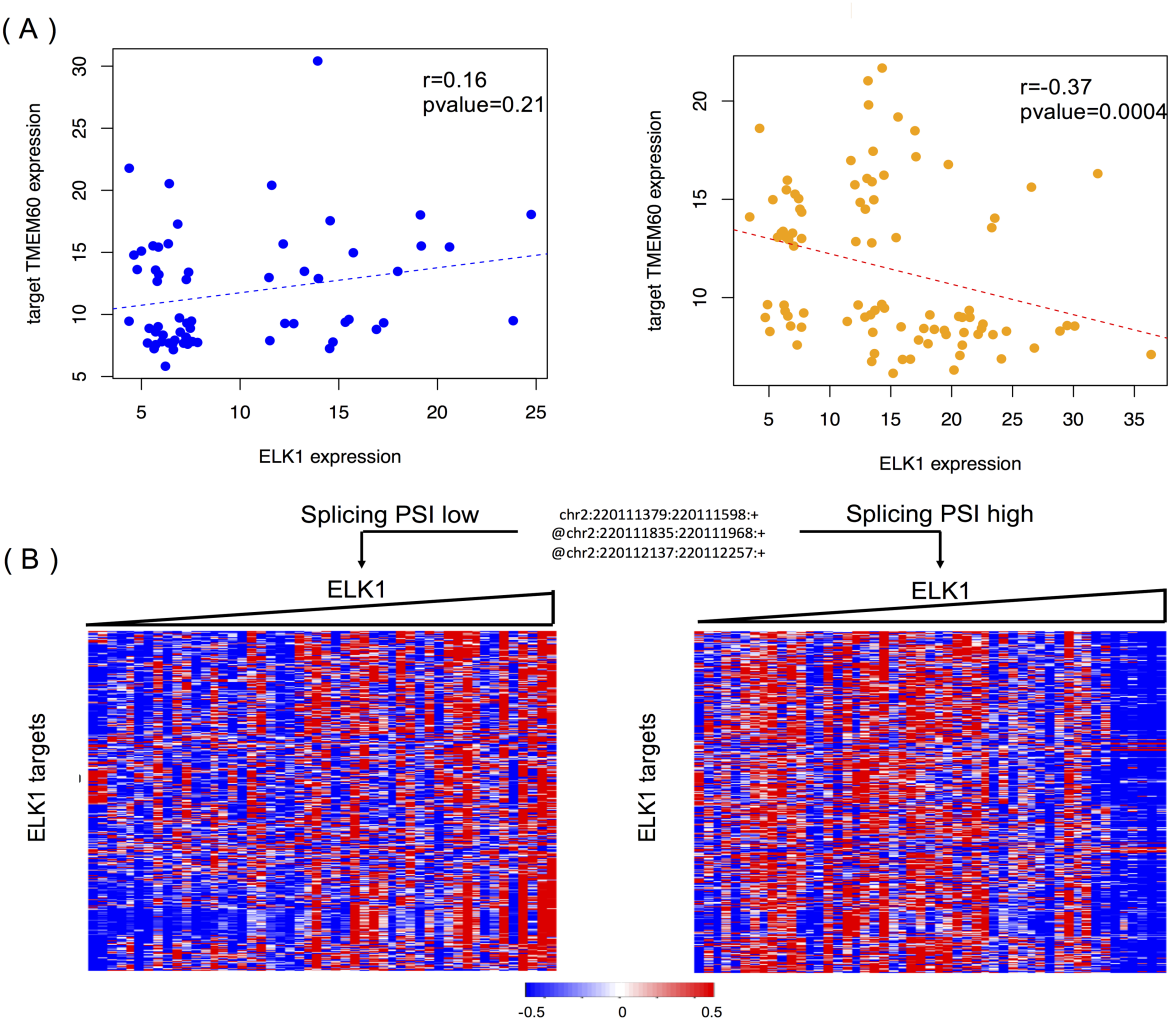
STK16 is a modulator that affects the transcriptional activity of ELK1 in LGG. (A) Examples of differential regulation activities of ELK1 on its target under the modulation of STK16 with differential splicing levels. The spliced exon is excluded in the final production of STK16 in the first scenario, and the exon is included in the final production of STK16 in the second scenario. (B) STK16 regulates the stability of ELK1. See Fig. 3B for interpretation of this graph.

Figure 6A shows the modulating effect of STK16 on ELK1, and TMEM60 is one of targets we randomly detected. The samples in the two groups are selected using the same method mentioned above. A negative correlation (−0.37, *p* = 0.0004) is only shown when the exon is included in the final product. The exon’s impact in protein function analysis (Li et al., 2017b) shows that this alternatively spliced exon has a high disease probability of 0.67, which indicates that changes in the exon inclusion or exclusion ratio might cause a gain or loss in protein function. The specific alternatively spliced exon of STK16 encodes a kinase domain, and thus it is not surprising that the loss of this exon will cause a change in protein function and may ultimately influence numerous normal gene functions.

Figure 6B shows the global modulating effect of STK16 on ELK1. The low and high inclusion groups contain samples with the top and bottom 30% of PSI values, which indicate exon exclusion and inclusion in the final protein. A positive correlation between ELK1 and its targets is clearly shown when the exon is excluded, whereas this correlation becomes negative when the exon is included. This result suggests that the 4th cassette exon in STK16 is important to final protein function and that changes in the splicing level of STK16 are associated with the differential transcriptional activity of ELK1.

## Discussion

In this work, a known probabilistic method, GEM, was used to infer alternative splicing-modulated triplet relationships (Babur et al., 2010). The type of input data was gene expression, and the method was built for discovering modulated regulation relationships based on gene expression levels. Our preliminary work (Li et al., 2017a) built a regression model to infer similar triplet relationships, but we focused on gene expression and the splicing level. We changed the input data of GEM to gene expression and alternative splicing values and compared the results of the two methods for discovering modulated triplets. The results showed that numerous overlapping triplets could be detected. While our previous method has higher sensitivity, the GEM method is much more robust. Another reason we used modified GEM is that the method classifies the modulator regulation mode into six categories, which is helpful for understanding the modulation mechanisms.

Several alternative splicing isoform outcome-modulated triplets were detected in this study. The most important TF we discovered was ELK1, whose activity was impacted by many alternative splicing isoform outcomes, such as STK16 and APP. Previous studies have shown that ELK1 phosphorylation can be modulated in various central nervous system diseases (CNS diseases), such as Alzheimer’s’ disease, Huntington’s disease, Down syndrome and depression (Besnard et al., 2011; Demir et al., 2011). The inferred modulator STK16 is a Ser/Thr kinase, and STK16 exhibited protein kinase activity in both in vitro and in vivo kinase assays. Some kinases, such as casein kinase II, have been reported to interact with TFs (Yamaguchi et al., 1998), but how those kinases regulate the transcription machinery remains unclear. In this study, we identified the fourth alternative spliced exon as important for STK16. The fourth exon is located in a region that encodes a kinase domain named Pkinase. Hence, the STK16 splice isoform with the kinase domain may play a vital role in regulating transcription activity.

A preliminary study showed that STK16 exhibits an autophosphorylation pattern. STK16 also phosphorylates MBP and histone H1, both of which are often used as substrates for Ser/Thr kinases, as well as the recombinant His-tagged ELK1 activation domain (Ohta et al., 2000). The N-terminal third and central portions of STK16, which contain residues essential for Ser/Thr kinase activity, are required for its transcriptional regulatory function and therefore for DNA-binding ability (Besnard et al., 2011). Hence, different splicing outcomes are associated with the phosphorylating activity of STK16 and may influence the interaction with TFs. Other preliminary studies have reported that STK16 strongly enhances the ELK1 signal, suggesting that STK16 is involved in the phosphorylation of some nuclear TFs. STK16 could activate the MAP kinase pathway, leading to ELK1 activation, similar to the TGF-β signaling pathway. As the MAP kinase pathway has been reported to be involved in one of the TGF-β signaling pathways (Hartsough & Mulder, 1995; Reimann et al., 1997), STK16 may be a candidate linking the MAP kinase pathway to TGF-β signaling. This may explain why STK16 could regulate the transcriptional activity of ELK1.

Another modulator, APP, encodes a cell surface receptor and transmembrane precursor protein that is cleaved by secretases to form a number of peptides. Some of these peptides are secreted and can bind to the acetyltransferase complex APBB1/TIP60 to promote transcriptional activation, while others form the protein basis of the amyloid plaques found in the brains of patients with Alzheimer’s disease. Mutations in this gene have been implicated in autosomal dominant Alzheimer’s disease and cerebroarterial amyloidosis. Multiple transcript variants encoding several differential isoforms have been identified for this gene. By searching the Integrated Interactions Database (IID) and restricting the interaction partners to only those supported by experimental evidence, we obtained protein-protein interaction evidence for APP and ELK1.

One splicing event of APP detected as a modulator was “chr21:27354657:27354790:- @ chr21:27372330:27372497:-@chr21:27394156:27394358:-”. The alternatively spliced exon was the seventh exon, which contains a vital domain named BPT/Kunitz inhibitor (BPTI). The splice isoforms that contain the BPTI domain possess protease inhibitor activity that induces an AGER-dependent pathway involving activation of p38 MAPK, resulting in internalization of amyloid-beta peptide and leading to mitochondrial dysfunction in cultured cortical neurons. ELK1 phosphorylation has been shown to be modulated in various CNS diseases (Besnard et al., 2011). Hence, we conclude that the detected splice isoform of APP with the BPT1 domain could influence the transcriptional activity of ELK1.

A previous study reported that Abeta, ELK1, PS1 and APP are associated. Tong et al. (2004) showed that sublethal concentrations of Abeta interfere with BDNF-induced activation of ELK1 in cultured cortical neurons and result in altered SRE-driven gene regulation, which is likely to account for increased neuronal vulnerability. Pastorcic and Das (Pastorcic & Das, 2003) defined ELK1 as a potent repressor of transcription of the presenilin 1 gene (PS1), which encodes a protein required for the final protein (APP) that produces highly amyloidogenic variants of Abeta. PSI is genetically linked to the majority of cases of early-onset familial Alzheimer’s disease (FAD). Collectively, these data highlight the intriguing link connecting Abeta, ELK1, PSI and APP and also show that APP is associated with the activity of ELK1.

## Conclusions

We globally dissected the role of AS in regulating the transcriptional activity of TFs in LGG using TCGA-LGG data. ELK1, a member of the Ets oncogene family of TFs that functions in neurons, was one of the key TFs discussed in detail in this study. Two significant modulators, APP and STK16, were identified. The results showed that different alternatively spliced isoforms of APP/STK16 are associated with the transcriptional activity of ELK1 and play dual roles in CNS diseases. The presented results provide important insights on the modulating role of AS in transcription regulation in LGG as well as the role of signaling modules in neuronal survival in neurodegenerative processes.

##  Supplemental Information

10.7717/peerj.4686/supp-1 Supplemental Information 1 Table S1 & Table S2 Inferred APP and STK16 modulated triplets and their modulation categories.

10.7717/peerj.4686/supp-2 Supplemental Information 2 Raw code This file recodes the code we used to process raw data in this study.

10.7717/peerj.4686/supp-3 Supplemental Information 3 Raw data This raw data is the splicing profile of all splicing events.

10.7717/peerj.4686/supp-4 Supplemental Information 4 Raw data Lis of all the targets information of all the transcription factors.

10.7717/peerj.4686/supp-5 Supplemental Information 5 Raw data List of all the candidate splicing events of ELK1.

10.7717/peerj.4686/supp-6 Supplemental Information 6 Raw data List of all the targets of ELK1.

10.7717/peerj.4686/supp-7 Supplemental Information 7 Raw data The whole gene expression profile is too big to submit. So we separate it into five sub-files. This is the first part of gene expression profile.

10.7717/peerj.4686/supp-8 Supplemental Information 8 Raw data The second part of gene expression profile.

10.7717/peerj.4686/supp-9 Supplemental Information 9 Raw data The third part of gene expression profile.

10.7717/peerj.4686/supp-10 Supplemental Information 10 Raw data The fourth part of gene expression profile.

10.7717/peerj.4686/supp-11 Supplemental Information 11 Raw data The fifth part of gene expression profile.

## Additional Information and Declarations

### Data Availability

The following information was supplied regarding data availability: The raw data are provided in the Supplemental Files.
